# Afghan women and access to health care in the past 25 years

**DOI:** 10.1016/j.eclinm.2021.101235

**Published:** 2021-12-13

**Authors:** Zainab Ezadi, Nesa Mohammadi, Roqia Sarwari, Shakardokht M Jafari

**Affiliations:** a Midwifery faculty, Khatam al Nabieen University, Kabul, Afghanistan; b Tetra Tech, Kabul, Afghanistan; c Portsmouth Hospitals University NHS Trust, Portsmouth, UK; d Centre for Nuclear & Radiation Physics, University of Surrey, Guildford, UK

Over the past two decades, Afghanistan has received international support and assistance to fund its essential services. With the decline of support and aid in the past 7 years, and withdrawal acceleration following President Biden's announcement that US and NATO troops should leave Afghanistan, the lives and wellbeing of many Afghan girls and women were threatened as the Taliban took control of the country on August 15, 2021.^1^

Before this latest upheaval, women in Afghanistan were already facing great challenges in meeting their reproductive and sexual health needs. In the early 2000s, after decades of conflict, poverty, lack of health services, and births without doctors or midwives, Afghanistan had the second highest maternal mortality rate in the world. Many women were not able to access health care, and could only be examined by female health professionals as male doctors were not allowed to visit them.^2^ In late 2001, after the defeat of the Taliban regime, the Afghan Government and international donors prioritised the development of an effective health-care system, including expanding access to primary health care throughout the country. These efforts have led to significant gains, including a reduction in maternal mortality from 1450 maternal deaths in 2000 to 638 in 2017.^3^ Also, the proportion of births supervised by specialists increased from 12·4% in 2000 to 58·8% in 2018.^4^ Access to modern family planning services increased from 10% to 22% from 2003 to 2010^5^ and the United Nations estimated that the maternal mortality rate of 1100 per 100 000 live births in 2000 had fallen by 64% to 396 per 100 000 live births by 2015.^6^ However, as the USA and NATO decided on withdrawal in 2010 and international support declined, the health-care system deteriorated. According to a 2018 World Bank report,^7^ between 2004 and 2010, health-care services showed major improvements in Afghanistan, while in the period between 2011 and 2016, improvements slowed down.

Afghanistan Analysts Network reported in 2019 that health care across the country was generally poor.^8^ In a study conducted in 2018 on evolving public perceptions,^9^ when asked to name the biggest problems facing women in their local area, respondents most frequently cited illiteracy and lack of educational opportunities, followed by limits on women's rights that reduce their public participation and access to justice. Other challenges mentioned were the lack of employment opportunities and violence against women—predominantly domestic. Despite advances in gender equality over the past two decades post-Taliban regime, according to the 2020 Human Development Index, Afghanistan ranked 169th in terms of gender inequality, with reproductive health, women's empowerment, and economic activity reflecting high levels of gender inequality. Women also had difficulty accessing health care due to the high costs in transportation and medicines. The advances made in some indicators after withdrawal of international support in 2014 were stagnating or even mostly reversing, such as prenatal care and the presence of a professional at birth.^10^

The country's current instability could further increase poverty, affecting all communities and particularly the most vulnerable. The position of women in Afghanistan has changed substantially throughout the country's history; women have been deprived of their basic rights, as well as access to education, health care, and employment.^2^ Further disruption of women's education and training could also result in a long-term shortage of female health-care staff in the country. Despite two decades of effort, the provision of health care to women after the arrival of the Taliban in August 2021 could be at a lower standard than that expected internationally and nationally.^1^

Reports from the past 25 years on women's access to health care in Afghanistan largely focused on restrictions caused by insecurity and conflicts. However, poverty, traditions, inadequate health facilities, and lack of education are also major barriers. Given that half of the people of Afghanistan live below the poverty line, and so many additional barriers exist, health care is still out of reach for many Afghan women. To improve health care for the women of Afghanistan, each individual barrier and hindrance to women's health must be addressed.

## Declaration of Interests

None.
